# Dopant Evolution in Electrocatalysts after Hydrogen Oxidation Reaction in an Alkaline Environment

**DOI:** 10.1021/acsenergylett.3c00842

**Published:** 2023-07-14

**Authors:** Su-Hyun Yoo, Leonardo Shoji Aota, Sangyong Shin, Ayman A. El-Zoka, Phil Woong Kang, Yonghyuk Lee, Hyunjoo Lee, Se-Ho Kim, Baptiste Gault

**Affiliations:** † Max-Planck Institut für Eisenforschung GmbH, 40237 Düsseldorf, Germany; ‡ Department of Materials, Imperial College London, SW7 2AZ London, United Kingdom; § Department of Chemical and Biomolecular Engineering, Korea Advanced Institute of Science and Technology (KAIST), Daejeon 34141, Republic of Korea; ⊥ Fritz-Haber-Institut der Max-Planck-Gesellschaft, Berlin 14195, Germany; ¶ Department of Materials Science and Engineering, Korea University, Seoul 02841, Republic of Korea

## Abstract

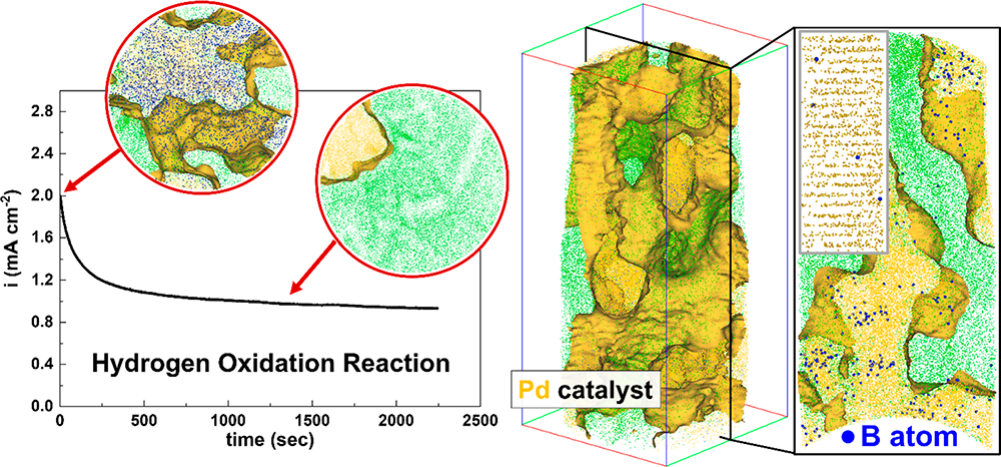

Introduction of interstitial dopants has opened a new pathway to optimize nanoparticle catalytic activity for, e.g., hydrogen evolution/oxidation and other reactions. Here, we discuss the stability of a property-enhancing dopant, B, introduced through the controlled synthesis of an electrocatalyst Pd aerogel. We observe significant removal of B after the hydrogen oxidation reaction. *Ab initio* calculations show that the high stability of subsurface B in Pd is substantially reduced when H is adsorbed/absorbed on the surface, favoring its departure from the host nanostructure. The destabilization of subsurface B is more pronounced, as more H occupies surface sites and empty interstitial sites. We hence demonstrate that the H_2_ fuel itself favors the microstructural degradation of the electrocatalyst and an associated drop in activity.

In a recent review article, Pérez-Ramírez et al. stated that “Catalysts are not immortal”, emphasizing that all catalysts degrade over time when subjected to chemical, electrical, or thermal stimuli,^1^ yet they are indispensable to enable the upcoming hydrogen economy. The most critical and expensive component of fuel cells, which can convert hydrogen gas to electricity with low cost,^2^ is the catalyst, accounting for more than 50% of the total stack cost.^3^ The large-scale application of fuel cells is limited by the degradation of electrocatalysts,^4,5^ however, as pointed out recently, most research is focused on the catalyst’s performance at all cost,^6,7^ and not on, e.g., the deactivation mechanisms that could help design more robust catalysts.

In this context, hydrogen-based fuel cells, including polymer electrolyte membrane,^8^ solid oxide,^9^ phosphoric acid fuel cells,^10^ and specifically anion exchange membrane fuel cells (AEMFCs) have attracted considerable attention.^11,12^ Especially, AEMFCs operate under alkaline conditions, allowing the use of inexpensive transition-metal catalysts on the cathode side, where the oxygen reduction reaction occurs, whereas on the anode side, electricity is generated from hydrogen through hydrogen oxidation reaction (HOR).

In general, Pd exhibits a substantially low catalytic activity for HOR in alkaline media (0.05 mA cm^–2^_Pd_).^13^ However, the performance of Pd-based catalysts has been improved by decorating the surface with Ir^14^ and Ru^15^ clusters, alloying with Ni,^16^ coating on a Cu nanowire substrate,^17^ and adding CeO_2_.^18^ Among the variants, the Pd-CeO_2_ catalyst, which exhibits the highest anode performance (54.5 mA cm^–2^_Pd_), has emerged as one of the most promising electrocatalysts in HOR.^19^ In our previous research,^20^ we demonstrated that an optimal level of B-doping can enable a delicate balance between the binding energies of H and OH, thereby leading to the development of an enhanced Pd-based catalyst for alkaline HOR through a simple NaBH_4_ reduction synthesis. Nevertheless, commercial implementation of these approaches has yet to be realized due to its low stability during operation. To address this challenge, the mechanisms by which hydrogen attacks and dissolves additives or dopants within electrocatalyst must be systematically investigated.

Here, we designed a model B-doped Pd catalyst to investigate the degradation mechanism during HOR. We synthesized Pd nanocatalysts using sodium borohydride (NaBH_4_) as a reducing agent in an aqueous solution with controlled kinetics so as to introduce over 2 at. % of B-dopants in Pd using the approach that we introduced in refs (21 and 22). Figure 1a–c shows scanning electron microscopy (SEM), transmission electron microscopy (TEM), and a three-dimensional (3D) reconstruction of an atom probe tomography (APT)^23,24^ analysis of the as-synthesized Pd nanoparticles, respectively. For APT measurement, the Ni matrix was used to encapsulate the porous catalyst following the protocol outlined in refs (25 and 26). From the microscopes, the Pd catalyst shows an aerogel-like network morphology with a ligament size of approximately 10 nm, and in the atom map, the iso-compositional surface (yellow) of Pd at 25 at. % highlights the Pd catalyst surface (see Figure 1c(i) and (ii)). Consistent with our previous work,^21,22^ a substantial amount (2.88 at. %) of B dopants from the NaBH_4_ reducing agent has been introduced in the Pd.

**Figure 1:**
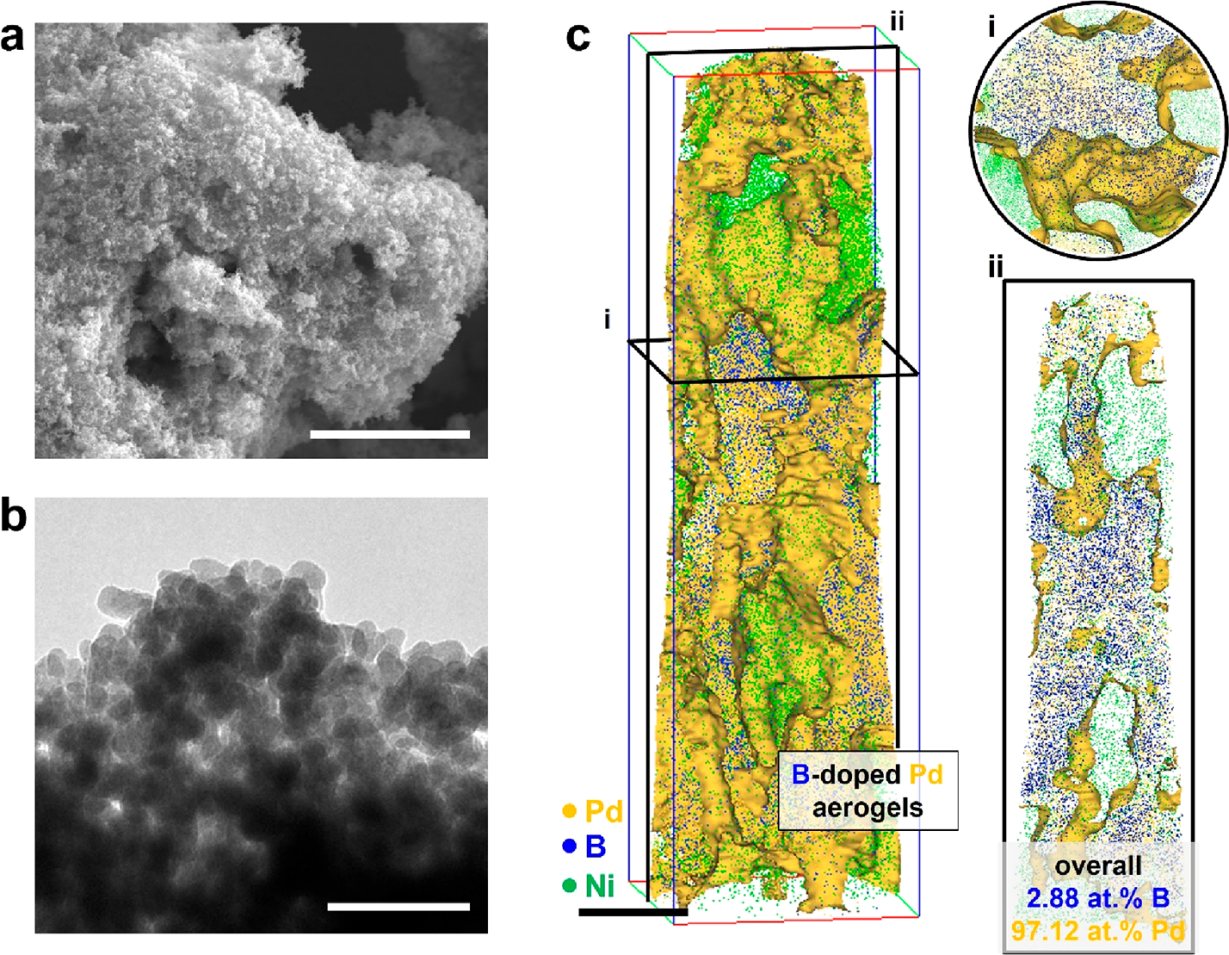
(a) SEM and (b) TEM images of the as-synthesized Pd aerogels. Scale bars are 5 μm and 50 nm, respectively. (b) 3D atom map of Pd aerogels embedded in Ni matrix. Yellow, blue, and green dots represent the reconstructed Pd, B, and Ni atoms, respectively. (i) and (ii) show 2 nm-thick sliced tomograms from the 3D map. Scale bar is 20 nm.

To investigate the changes in catalytic activity, the HOR performance of the B-doped Pd catalyst was evaluated in 0.1 M KOH electrolyte, using two electrochemical techniques, linear sweep voltammetry (LSV) and chronoamperometry.^22^ As shown in Figure 2a, the performance of the B-doped Pd catalyst rapidly decreased as the LSV was measured, and a certain performance was reached in the 10th measurement. Also, it was observed that the hydrogen oxidation current decreased in the chronoamperometry curve measured at 0.3 V (vs RHE). The current density decreases from 2.2 to 1.7 mA cm^–2^ after an operation time of 3600 s, which provides evidence of the catalyst’s degradation (see Figure 2b). In other words, the B-doped Pd catalyst was unstable in a H_2_-saturated HOR environment in alkaline solution. As a control experiment, we conducted the LSV and chronoamperometry curves using a B-undoped pristine Pd catalyst (Figure S1). Pristine Pd catalyst exhibited no decrease in hydrogen oxidation current during LSV and chronoamperometry measurement. Figure 2c and 2d shows the reacted B-doped Pd catalyst after HOR in an alkaline condition. The morphology of the catalyst was similar to its pristine state, and ligaments could still be observed.

**Figure 2:**
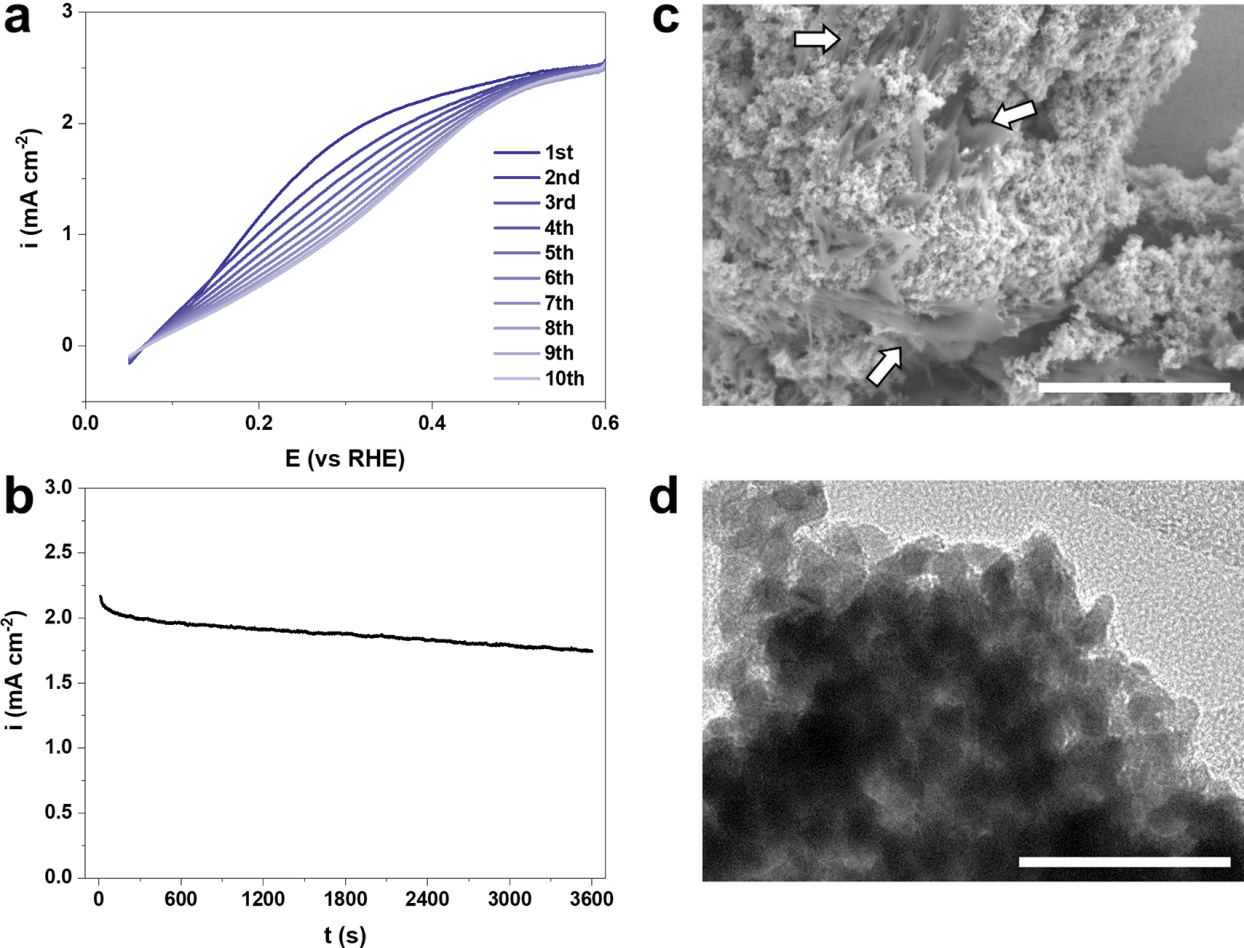
(a) HOR LSV curves of the B-doped Pd catalyst. LSV curves were repeatedly measured 5 times with a scan rate of 10 mV s^–1^ from 0.6 to 0.05 V (vs RHE). (b) Chronoamperometry curve measured at 0.3 V (vs RHE). HOR performance of catalyst was evaluated in H_2_-saturated 0.1 M KOH electrolyte using 3-electrode half-cell setup. (c) SEM and (d) TEM images of post-HOR Pd catalysts. Scale bars are (up) 5 μm and 50 nm (down). Noticeably, a part of the aerogel’s surface was covered with a dried salt compound from the electrolyte (indicated by a white arrow in panel c).

To investigate the chemical evolution of the post-HOR catalyst, we collected the Pd particles and prepared them for APT measurement, as shown in Figure 3. The atomic composition of B decreased more than 12-fold, down to only 0.225 at. %. After prolonged electrocatalytic use, the B atoms appear to have etched away from the Pd catalyst, accompanying the decreased catalytic activity. Figure 3a reveals even regions in which no B atom was measured. In other data sets, the remaining B atoms appeared in the form of clusters inside the Pd but inhomogeneously segregated (Figure 3b and 3c). It is worth noting that the leaching of subsurface B atoms within B-doped Pt-group nanoparticles under operando electrocatalytic conditions can be mitigated by constructing ordered intermetallic borides, thereby improving structural stability while maintaining catalytic activity.^27^ The structure, after the electrochemical test, was still in a crystalline phase as clear atomic lattices of fcc Pd were observed from the reconstructed aerogel’s grain (see inset in Figure 3c). The Pearson coefficients of the frequency distribution of B (*r*_B_) for the pre- and postcycled Pd were compared to measure the degree of randomness/segregation,^28^ taking values of 0.21 and 0.66, respectively, indicating that B went from close to random (*r*_B_ = 0) to close to fully segregated (*r*_B_ = 1) upon HOR.

**Figure 3:**
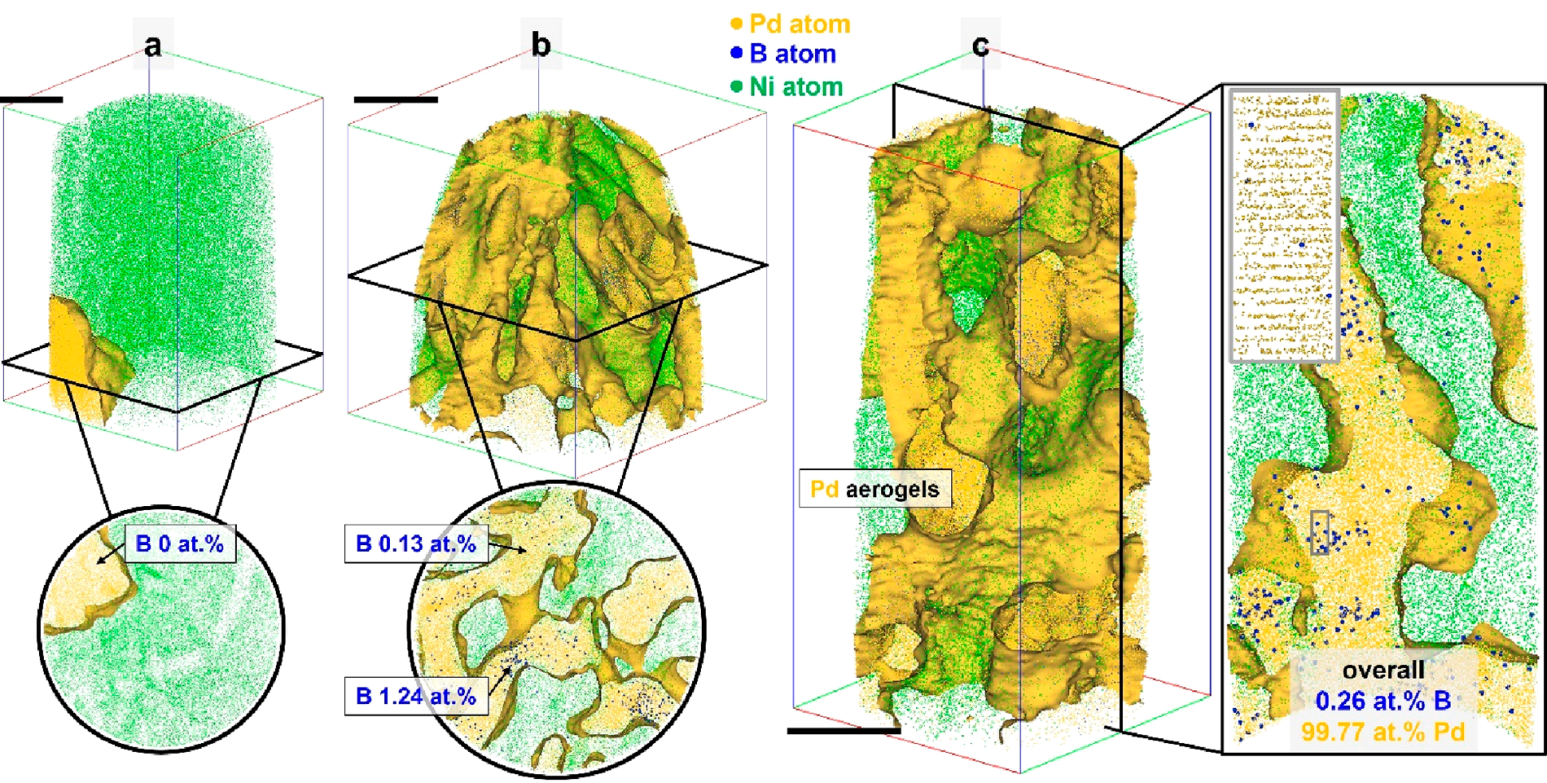
APT results of three different specimens of post-HOR Pd catalysts: Pd (a) without and (b, c) with B. All scale bars are 20 nm.

In HOR conditions, the B-doped Pd catalyst surfaces were continuously exposed to H_2_ gas, which results in numerous H_2_ dissociation and adsorption events.^29^ To understand how B dopants are affected by the presence of hydrogens, we used density-functional theory (DFT) to calculate the boron binding energies [*E*_b_(B)] on Pd(111) surfaces as a function of H coverage (Θ_H_) and their electronic structures, as shown in Figure 4 (see Supporting Information for computational details, Figure S2). It is noted that Θ_*i*_ is defined as a ratio between the number of adsorbates *i* and host Pd atoms in a monolayer (ML) unit, and negatively larger *E*_b_ indicates stronger binding. Having known that interstitial-octahedral sites (B_octa_) are the most favorable for B dopants over other high-symmetric binding sites [e.g., surface fcc site (B_fcc_)],^22^ we performed further calculations. First, *E*_b_(B_octa_), as shown in Figure 4a, gradually gets weaker by up to 0.84 eV/B in the regime below 1 ML of Θ_H_ where hydrogens adsorb only on the Pd surface (blue lines), whereas *E*_b_(B_fcc_) gets slightly stronger (orange lines). Such a reduction in *E*_b_(B_octa_) for 0.25 ML B_octa_ is small enough to keep B_octa_ still more stable than B_fcc_. The weak binding for the 1 ML systems implies the instability of both B_octa_ and B_fcc_ (blue and orange dashed lines, respectively), which could be understood by large distortion of surface structures, strong repulsive interactions between B atoms, and changes in d-band centers due to the large concentration of B atoms.^20^ However, assuming that the 1 ML models would represent highly doped nanoparticles, we focus on the models with lower coverage (i.e., 0.25 ML) to explain our observations in the experiments. Considering the possibility that the smallest element, hydrogens, can easily penetrate into subsurface regions,^30,31^ we model one case where hydrogens adsorb only at the first subsurface sites (purple line), which shows a twice larger reduction in *E*_b_(B_octa_) compared to the case of surface hydrogens. Furthermore, to elucidate the impact of subsurface hydrogens on *E*_b_(B), we additionally model the regime beyond 1 up to 2.75 ML by allowing hydrogen penetration to the deeper subsurface, which leads to a reverted order between B_octa_ and B_fcc_ at 2.75 ML. This implies that subsurface binding is no longer energetically more stable than surface binding in H-rich conditions.

**Figure 4:**
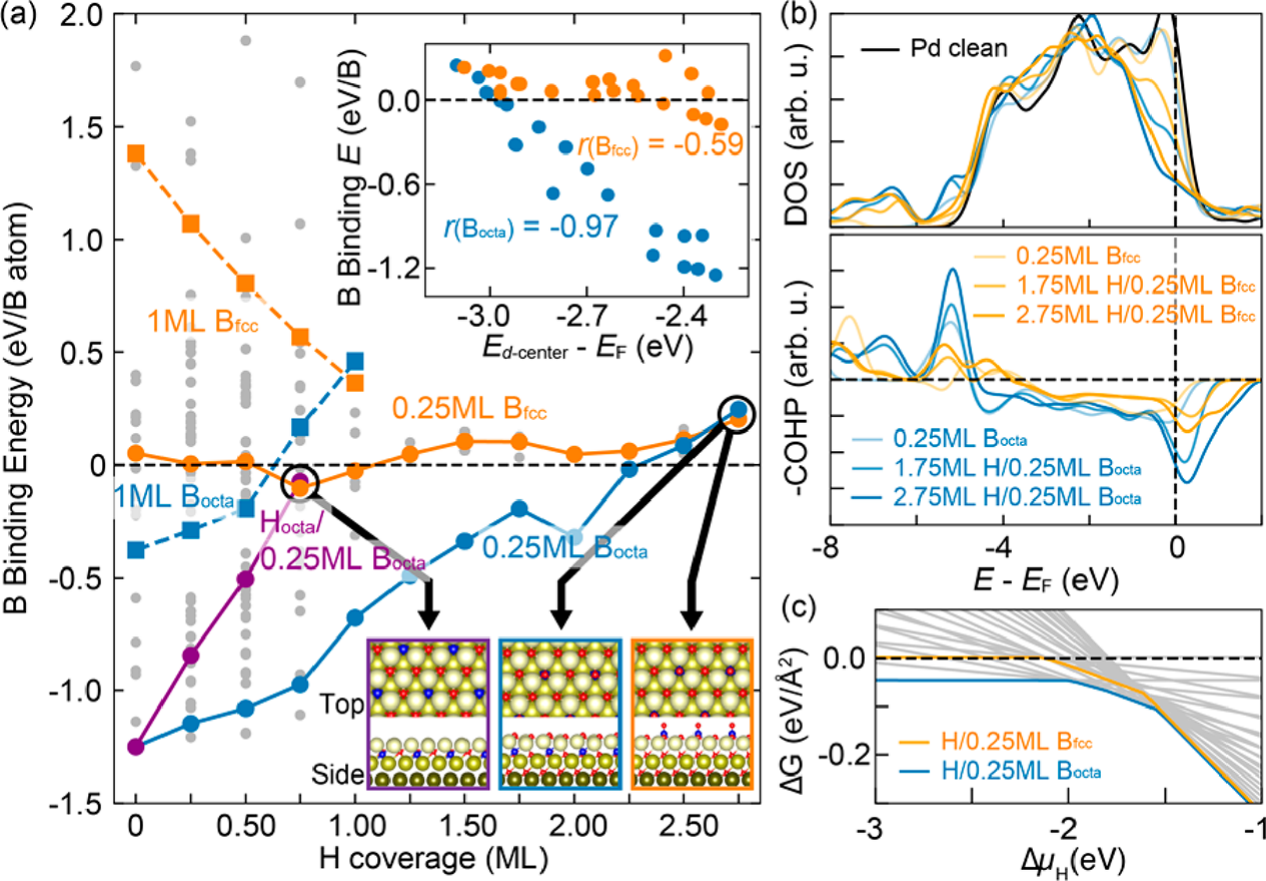
(a) *E*_b_ as a function of Θ_H_. The blue (orange) lines indicate *E*_b_ for the structures where hydrogens adsorb from the surface to subsurface sites up to 2.75 ML with B at octahedral (fcc) sites. The solid (dotted) lines are for Θ_B_ = 0.25 ML (1 ML). The purple line and the gray points represent *E*_b_ for the structures where hydrogens occupy only 1st subsurface sites, and for other models with B at the different sites, respectively. The right top and bottom insets depict the correlation plot between *E*_*d*-center_ and *E*_b_, and three models have the weakest B binding in the presence of subsurface hydrogens. Red, blue, and yellowish balls represent hydrogen, boron, and palladium atoms, respectively. See Supporting Information for other models. (b, top) Density-of-states (DOS) for *d*-states of the outmost three Pd layers and (bottom) COHP values for the Pd–B bonds in the selected models. (c) Gibbs free energies as a function of H chemical potential for all structures with 0.25 ML of Θ_B_. The energetically most stable lines are depicted as colored lines and the rest as gray lines.

To further understand the origin of the destabilization of subsurface B dopants, we performed electronic structure analyses. The Pd *d*-band center (*E*_*d*-center_) and its scaling relations^32,33^ with *E*_b_ are shown in the top-right inset of Figure 4a. Pearson coefficient of −0.97 for the B_octa_ cases indicates that *E*_b_(B_octa_) is closely affected by Pd *d*-states compared to that for B_fcc_ (−0.59 for B_fcc_). We additionally find that Pd *d*-states near the Fermi energy (*E*_F_) are largely redistributed due to the formation of Pd–H bonds, as illustrated in the density-of-states (DOS) at the top panel of Figure 4b. Furthermore, the crystal orbital Hamilton population (COHP) analysis at the bottom panel demonstrates that the population of antibonding states of Pd–B bonds near *E*_F_ more increases for B_octa_ than for B_fcc_ with an increase of subsurface hydrogens. This array of data allows us to conclude that hydrogens underneath surfaces cause a redistribution of Pd *d*-states, a reduction in the *d*-band center, and filling more antibonding states of the Pd–B bonds for B_octa_ than those for B_fcc_. Thus, they eventually bring stronger destabilizations of B dopants in Pd structures. In fact, the relative Gibbs free energy for both 0.25 ML B_octa_ and B_fcc_ with respect to the adsorbate-free surface, as shown in Figure 4c, shows that energy difference between B_octa_ and B_fcc_ becomes within only 5 meV/Å^2^ at H-rich conditions.

Therefore, exposure to the H-rich condition does not guarantee the superior stability of B_octa_ over B_fcc_ anymore, which would lead to a segregation of B dopants to surfaces. It is important to note that we do not consider either the effect of the particular choice of B chemical reservoir in solution (e.g., B-related ions) or that of the applied bias under HOR onset potentials. Considering the fact, however, that the negative electrode potential during HOR reactions combined with a specific B-related reservoir in solution could weaken adsorbates bindings, the same process iteratively occurs when surface B adsorbates leave the host structure to be leached out, which explains dissolution of B dopants observed from APT.

The presence of dopants inside electrocatalysts influences their catalytic activity, including for alkaline-HOR. Here, although B-doping was shown to increase the performance, we have observed substantial degradation of the B-doped Pd electrocatalyst during HOR, which is explained by a preferential dissolution of the B dopants under the reaction conditions. Despite the relatively strong stability of subsurface B in Pd, *ab initio* DFT calculations predict that both the adsorbed and absorbed H lead to a significant decrease of the B stability in Pd, which makes it leave the host structure as the preferred scenario. Hence, H_2_ itself is a clear retardant of the HOR activity, as it accelerates the dissolution of the property-enhancing elements added to the electrocatalysts. These insights provide hints that only increasing the catalytic activity/kinetics is not sufficient, but it must also be understood how to prevent degradation of the catalyst by the H_2_ fuel to obtain truly superior HOR catalysts.

## Supporting Information Available

Materials and methods, sample preparations, advanced characterizations, computational calculation, additional electrochemical data (PDF)
