# Supplementary material for: Dopant Evolution in Electrocatalysts after Hydrogen Oxidation Reaction in an Alkaline Environment
Source: ACS Energy Lett. 2023 Jul 14;8(8):3381–6. doi: 10.1021/acsenergylett.3c00842 (PMC10425978; doi:10.1021/acsenergylett.3c00842)
Supplement: Supplementary file 1 — nz3c00842_si_001.pdf [file nz3c00842_si_001.pdf]

# Dopant evolution in electrocatalysts after hydrogen oxidation reaction in alkaline environment

Su-Hyun Yoo[a,b,†], Leonardo Shoji Aota[a,†], Sangyong Shin[c,†], Ayman El-Zoka[b], Phil Woong Kang[c], Yonghyuk Lee[d], Hyunjoo Lee[c], Se-Ho Kim[a,e,*], Baptiste Gault[a,b,*]

[a] Max-Planck Institut für Eisenforschung GmbH, 40237 Düsseldorf, Germany

[b] Department of Materials, Imperial College London, SW7 2AZ London, United Kingdom

[c] Department of Chemical and Biomolecular Engineering, Korea Advanced Institute of Science and Technology (KAIST), Daejeon 34141, Republic of Korea

[d] Fritz-Haber-Institut der Max-Planck-Gesellschaft, Berlin 14195, Germany

[e] Department of Materials Science and Engineering, Korea University, Seoul 02841, Republic of Korea

[†]co-first authors

[*]co-corresponding authors

# Table of Contents

*Experimental Section* ........ *2*
Pd nanocatalyst synthesis ........ 2
SEM analysis ........ 2
TEM analysis ........ 3
APT analysis ........ 3
Electrochemical Measurements for HOR in Alkaline ........ 3
*Computational Details* ........ *5*
Density-functional theory (DFT) calculations ........ 5
Thermodynamics and energetics ........ 8
Electronic structures ........ 9
*References* ........ *10*

## Experimental Section

### Pd nanocatalyst synthesis

B-doped Pd catalysts were synthesized following a wet-chemical borohydride reduction method[1]. First, each potassium tetrachloropalladate (99.99%, Sigma-Aldrich) and sodium borohydride (98.0%, Sigma-Aldrich) powder was separately dissolved in distilled water. The mole ratio of $BH_4$ ions to Pd ions was set to 10. Two as-prepared solutions were mixed, and after vigorous bubbling reaction, the Pd catalysts were synthesized. The catalysts were centrifuged to collect and were re-dispersed in distilled water for washing protocol. This process was done for three times to remove any surface remaining impurities.

### SEM analysis

After the synthesis, the catalysts were mounted on an Au electrode. Before the HOR/HER experiment, microstructural investigations were carried out using SEM (ZEISS Merlin) at an

acceleration voltage of 10 kV. Then the catalyst-mounted electrode was tested for HER/HOR and, after the experiment, the electrode was again analyzed with SEM.

TEM analysis

TEM analysis was performed using Talos F200X (FEI) instrument with an acceleration voltage of 200 kV. For a sample preparation, 1 mg of the as-synthesized catalyst was dispersed in 5 ml of ethanol and deposited onto a TEM grid (lacey carbon Cu 300 mesh grid). The grid was then dried overnight in a vacuum desiccator before conducting the TEM analysis. Following the electrochemical experiments, the catalyst deposited on the glassy-carbon electrode was re-dispersed in ethanol and drop-cast onto a TEM grid.

APT analysis

The pre-HOR Pd catalysts was encapsulated in a Ni film using co-electroplating method as described in Ref.[2]. Ga-ion sourced focused ion beam (Thermo Fischer Helios) was used to fabricate <100 nm radius APT specimens from the Pd nanoparticles/Ni composite film. APT measurements were carried out using Cameca LEAP 5000 XS system. To suppress a diffusion of the surface atoms between field evaporation sequences, the specimen temperature was set to cryogenic temperature of 60 K. A pulsed UV laser mode at a detection rate of 1%, a laser pulse energy of 50 pJ, and a pulse frequency of 125 kHz were set for data acquisition parameters.

Electrochemical Measurements for HOR in Alkaline

Electrochemical measurements were conducted at room temperature using a CHI 760e potentiostat. B-undoped Pd catalyst (99.9%, Sigma-Aldrich; <1 μm in size) were used as a control group. To prepare the catalyst ink, 5mg of the catalyst was dispersed in 2 ml of isopropyl alcohol along with 6.66μl of nafion (5 wt.% sol. Sigma Aldrich), which was then sonicated for 20 minutes. Subsequently, 7.5 μl of the ink was drop-casted on the glassy carbon disk Pt ring electrode (surface

area = 0.2472 $cm^2$). A double junction Hg/HgO filled with 1M KOH was used as the reference electrode, and a carbon rod was used as the counter electrode. Before half-cell tests, a reversible hydrogen electrode (RHE) was calibrated by interconversion point of hydrogen oxidation and evolution current in a high-purity $H_2$-saturated 0.1 M KOH solution using a Pt rotating disk electrode.[3] The as-prepared electrode was immersed in a 100 ml solution of 0.1 M KOH, which was purged with Ar gas for 30 minutes. To activate the catalyst surfaces, 50 cycles of cyclic voltammetry scans were performed at a scan rate of 50 mV $s^{-1}$ ranging from 0.4 V to 1.0 V (vs RHE) under Ar-saturated condition. The LSV curve for the hydrogen oxidation reaction (HOR) was obtained by performing linear sweep voltammetry from 0.6 V to 0.05 V (vs RHE) at a scanning rate of 10 mV $s^{-1}$. The LSV curves were measured consecutively for 10 cycles, with a 1-minute quiescent time provided before each LSV curve measurement. A sufficient amount of $H_2$ gas (100 sccm) was supplied to the electrolyte (100 mL) using a gas dispersion tube with fitted cylinders during the LSV measurements. The chronoamperometry curve for the HOR was measured for 1 hour at 0.3 V (vs RHE) for B-doped Pd catalyst and 0.5V (vs RHE) for B-undoped Pd catalyst. Both LSV and chronoamperometry curve measurement was conducted under $H_2$-saturated condition with a rotating electrode speed of 1600 rpm.

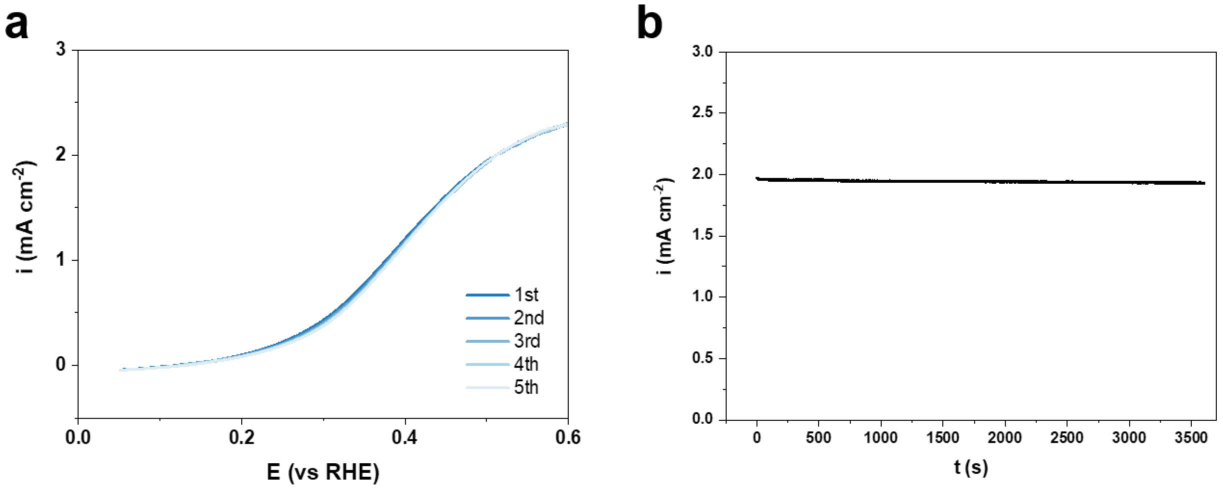

**Figure S1.** a) HOR LSV curves of B-undoped Pd catalyst. LSV curves were repeatedly measured 5 times with a scan rate of 10 mV $s^{-1}$ from 0.6 V to 0.05 V (vs RHE). (b) Chronoamperometry curve measured at 0.5 V (vs RHE). In the B-undoped Pd catalyst, no decrease in hydrogen oxidation current was observed during LSV and chronoamperometry measurements.

## Computational Details

Density-functional theory (DFT) calculations

The Vienna Ab-initio Simulations Package (VASP) code[4,5] was used for all DFT calculations employing the projector augmented wave (PAW) method[6]. Plane-wave cutoff energy of 500 eV was used. Electronic and ionic relaxations were carried out until the total energy convergence was less than $10^{-5}$ eV. The generalized gradient approximation (GGA) due to Perdew, Burke, and Ernzerhof (PBE) was used for the exchange-correlation functional[7]. A **Γ**-centered (8×8×8) *k*-points grid was used for face-centered cubic Pd bulk structures, and correspondingly folded grids were used according to the size of slab structures and their supercells. The implicit solvation model implemented in the VASP code by Mathew *et al.* (*i.e.*, VASPsol), where the solvent accessible surface was considered from the quantum mechanically calculated charge density of solute[8], was used with setting the dielectric constant of water, $\varepsilon = 80$, to include the impact solvent has on the energetics of adsorbate-substrate models.

A supercell containing a symmetric slab of 13 Pd atomic layers (ALs) with a thickness of 27.44 Å and a vacuum region of 18 Å was constructed for surface calculations. The three outermost ALs of the slab were relaxed, while the rest ALs were fixed at their bulk positions.

To construct B-doped Pd surface structures, following our previous work where B binding behavior was investigated[9], B dopants were positioned at surface binding sites [*i.e.*, top, bridge, face-centered cubic (fcc), hexagonal close-packed (hcp) sites] and sub-surface binding sites [*i.e.*, octahedral (octa) and two types of tetrahedral (tetra) sites] depending on its surface coverage in

differently sized slab structures. The surface coverage (Θ) was defined as the ratio between the number of adsorbate atoms and the surface of Pd atoms in the outmost surface layer. For H-adsorbed models, we constructed multiple structures considering their possible configurations for each coverage from 0 to 2.75 ML, and representative structures are shown in Figure S1. For $B_{octa}$ models, hydrogen adsorbates are positioned except for the subsurface sites $B_{octa}$ occupied due to their fully-coordinated environment. However, for $B_{fcc}$ models, we allow hydrogen adsorbates to be on top of the surface $B_{fcc}$ dopants, assuming that the formations of H-B bonds are inevitable under the hydrogen-rich condition.

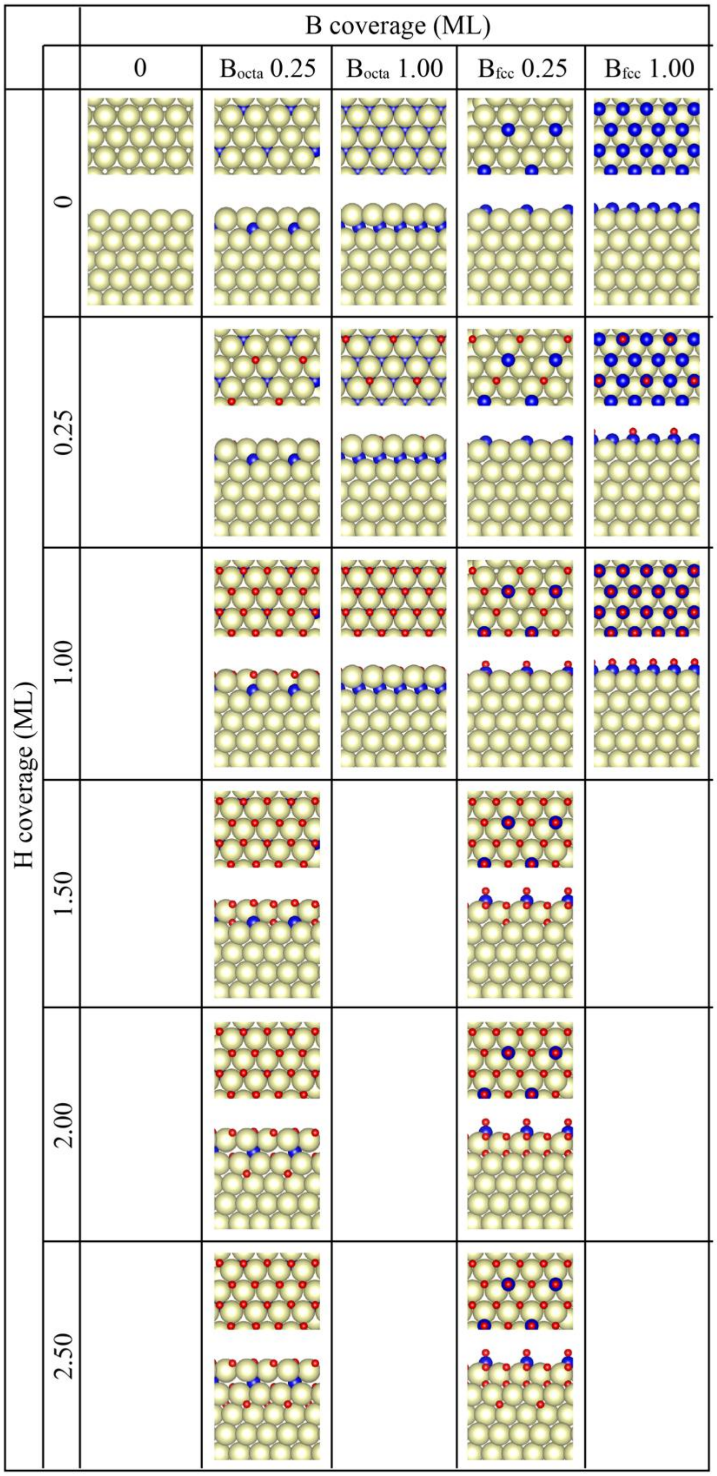


**Figure S2.** Representative surface models at the given coverages (ML) of hydrogen and boron atoms. In each panel, a top (side) view is shown on the top (bottom). Red, blue, and mild yellow balls represent hydrogen, boron, and palladium atoms, respectively. Please note that we show only one representative surface model for each coverage out of all calculated configurations.

Thermodynamics and energetics

Having known B-doped Pd surface structures, the impact of H adsorption on the binding behaviors of the B dopants was investigated by additionally constructing H-adsorbed B-doped Pd surface structures. First, H adsorbates were positioned at fcc surface binding sites, known as the thermodynamically most stable sites[10–16], depending on H surface coverage ($\Theta_H$), whereas H atoms are positioned atop site of B dopants for the cases where B dopants were positioned at surface binding sites. For the structures where B dopants were located at octahedral sites with $\Theta_B$ of 0.25 ML, two sets of H adsorbate-substrate models were additionally made to investigate the impact of the presence of sub-surface H adsorbates. On the one hand, H adsorbates were solely positioned at the rest of the sub-surface-octahedral sites up to 0.75 ML except for the site B dopants occupy without H surface adsorbates. On the other hand, H adsorbates were introduced at the rest of the sub-surface-octahedral sites to the structure with $H_{fcc}$ 1 ML and $B_{octa}$ 0.25 ML from 1.25 ML to 1.75 ML.

The binding energy ($E_b^B$) of B dopants as a function of H surface coverage was calculated as

$$E_{\mathrm{b}}^{\mathrm{B}}(\Theta_{\mathrm{H}}) = \frac{1}{2N_{\mathrm{B}}}\left(E_{\mathrm{tot}}^{\frac{\mathrm{H}}{\mathrm{B}}-\mathrm{Pd}}(\Theta_{\mathrm{H}}) - E_{\mathrm{tot}}^{\frac{\mathrm{H}}{\mathrm{Pd}}}(\Theta_{\mathrm{H}}) - 2N_{\mathrm{B}}\mu_{\mathrm{B}}\right)$$

where $E_{\mathrm{tot}}^{\mathrm{H/B-Pd}}$ and $E_{\mathrm{tot}}^{\mathrm{H/Pd}}$ are the DFT calculated total energies of the B-doped and B-free Pd surface structures with H adsorbates, respectively. $N_{\mathrm{B}}$ is the number of B dopants on one surface of the symmetric slab structure. $\mu_{\mathrm{B}}$ is the chemical potential of B dopants with respect to the relevant reference phase. In this work, the reference phase for B is chosen with the rhombohedral α-phase of B which is more stable than β-phase.

The relative Gibbs free energy of the surface phase α ($\Delta G_{\alpha}$) with respect to the adsorbate-free Pd surface was calculated as

$$\Delta G_{\alpha}(\mu_{\mathrm{H}}, \mu_{\mathrm{B}}) = \frac{1}{2A}\left(E_{\mathrm{tot},\alpha}^{\frac{\mathrm{H}}{\mathrm{B}}-\mathrm{Pd}} - E_{\mathrm{tot}}^{\mathrm{Pd\ clean}} - 2N_{\mathrm{B}}\mu_{\mathrm{B}} - 2N_{\mathrm{H}}\mu_{\mathrm{H}}\right)$$

where $E_{\mathrm{tot}}^{\mathrm{Pd\ clean}}$ is the DFT total energy of B- and H-free Pd surface. Considering the circumstance in which $H_2$ gas is continuously injected to the surface, $\mu_{\mathrm{H}}$ is evaluated with respect to the $H_2$ gas assuming T = 300K and $p$ = 1 atm, by including zero-point energy ($E_{\mathrm{ZPE}}^{\mathrm{H_2}}$)[17] and temperature- and pressure-dependent free energy contributions using the tabulated enthalpy ($H$) and entropy ($S$)[18] as follows,

$$\mu_{\mathrm{H}}(\Delta\mu_{\mathrm{H}}, T, p) = \frac{1}{2}\Big\{E_{\mathrm{tot}}^{\mathrm{H_2}} + E_{\mathrm{ZPE}}^{\mathrm{H_2}} + \left[H_{\mathrm{H_2}}(T, p^0) - H_{\mathrm{H_2}}(0\mathrm{K}, p^0)\right]$$

$$- T\left[S_{\mathrm{H_2}}(T, p^0) - S_{\mathrm{H_2}}(0\mathrm{K}, p^0)\right]\Big\} + k_B T \ln\frac{p}{p^0} + \Delta\mu_{\mathrm{H}}$$

Electronic structures

The d-band center is calculated by

$$E_{d-\mathrm{center}} = \frac{\int_{-\infty}^{E_{\mathrm{F}}} E * \mathrm{DOS}(E)}{\int_{-\infty}^{E_{\mathrm{F}}} \mathrm{DOS}(E)}$$

where $E$ is the eigen energy and DOS($E$) is the density-of-states of Pd $d$-states.

To understand detailed chemical bonding nature, the crystal orbital Hamilton population (COHP) analysis was performed using the LOBSTER code[19,20]. Specifically, the projected COHP pairs between B atom and neighboring Pd atoms are summed up where the bonding, nonbonding, and antibonding interactions between them can be distinguished.

## References


(1) Schlesinger, H. I.; Brown, H. C.; Finholt, A. E.; Gilbreath, J. R.; Hoekstra, H. R.; Hyde, E. K. Sodium Borohydride, Its Hydrolysis and Its Use as a Reducing Agent and in the Generation of Hydrogen. *J Am Chem Soc* **1953**, *75* (1), 215–219. https://doi.org/https://doi.org/10.1021/ja01097a057.

(2) Kim, S.-H.; Kang, P. W.; Park, O. O.; Seol, J.-B.; Ahn, J.-P.; Lee, J. Y.; Choi, P.-P. A New Method for Mapping the Three-Dimensional Atomic Distribution within Nanoparticles by Atom Probe Tomography (APT). *Ultramicroscopy* **2018**, *190*, 30–38. https://doi.org/10.1016/j.ultramic.2018.04.005.

(3) Niu, S.; Li, S.; Du, Y.; Han, X.; Xu, P. How to Reliably Report the Overpotential of an Electrocatalyst. *ACS Energy Letters*. American Chemical Society April 10, 2020, pp 1083–1087. https://doi.org/10.1021/acsenergylett.0c00321.

(4) Kresse, G.; Furthmüller, J. Efficient Iterative Schemes for Ab Initio Total-Energy Calculations Using a Plane-Wave Basis Set. *Phys. Rev. B* **1996**, *54*, 11169–11186.

(5) Kresse, G.; Furthmüller, J. Efficiency of Ab-Initio Total Energy Calculations for Metals and Semiconductors Using a Plane-Wave Basis Set. *Comput. Mater. Sci.* **1996**, *6*, 15–50.

(6) Blöchl, P. E. Projector Augmented-Wave Method. *Phys. Rev. B* **1994**, *50*, 17953–17979.

(7) Perdew, J. P.; Burke, K.; Ernzerhof, M. Generalized Gradient Approximation Made Simple. *Phys Rev Lett* **1997**, *78* (7), 1396. https://doi.org/10.1103/PhysRevLett.78.1396.

(8) Mathew, K.; Sundararaman, R.; Letchworth-Weaver, K.; Arias, T. A.; Hennig, R. G. Implicit Solvation Model for Density-Functional Study of Nanocrystal Surfaces and Reaction Pathways. *J Chem Phys* **2014**, *140* (8), 84106. https://doi.org/10.1063/1.4865107.

(9) Kim, S.-H.; Yoo, S.-H.; Shin, S.; El-Zoka, A. A.; Kasian, O.; Lim, J.; Jeong, J.; Scheu, C.; Neugebauer, J.; Lee, H.; Todorova, M.; Gault, B. Controlled Doping of Electrocatalysts through Engineering Impurities. *Advanced Materials* **2022**, *34* (28), 2203030. https://doi.org/https://doi.org/10.1002/adma.202203030.

(10) Kozlov, S. M.; Aleksandrov, H. A.; Neyman, K. M. Adsorbed and Subsurface Absorbed Hydrogen Atoms on Bare and MgO(100)-Supported Pd and Pt Nanoparticles. *J. Phys. Chem. C* **2014**, *118*, 15242.

(11) Paul, J. F.; Sautet, P. Density-Functional Periodic Study of the Adsorption of Hydrogen on a Palladium (111) Surface. *Phys. Rev. B* **1996**, *53*, 8015.

(12) L\ovvik, O. M.; Olsen, R. A. Adsorption Energies and Ordered Structures of Hydrogen on Pd(111) from Density-Functional Periodic Calculations. *Phys. Rev. B* **1998**, *58*, 10890.

(13) Dong, W.; Kresse, G.; Furthmüller, J.; Hafner, J. Chemisorption of H on Pd(111): An Ab Initio Approach with Ultrasoft Pseudopotentials. *Phys. Rev. B* **1996**, *54*, 2157.

(14) Roudgar, A.; Groß, A. Local Reactivity of Thin Pd Overlayers on Au Single Crystals. *Journal of Electroanalytical Chemistry* **2003**, *548*, 121–130. https://doi.org/https://doi.org/10.1016/S0022-0728(03)00230-4.

(15) Dong, W.; Hafner, J. ${\mathrm{H}}_{2}$ Dissociative Adsorption on Pd(111). *Phys Rev B* **1997**, *56* (23), 15396–15403. https://doi.org/10.1103/PhysRevB.56.15396.

(16) Conrad, H.; Ertl, G.; Latta, E. E. Adsorption of Hydrogen on Palladium Single Crystal Surfaces. *Surf. Sci.* **1974**, *41*, 435.

(17) Yoo, S. H.; Kim, S. H.; Woods, E.; Gault, B.; Todorova, M.; Neugebauer, J. Origins of the Hydrogen Signal in Atom Probe Tomography: Case Studies of Alkali and Noble Metals. *New J Phys* **2022**, *24* (1) 013008. https://doi.org/10.1088/1367-2630/ac40cd.

(18) Stull, D. R.; Prophet, H. *JANAF Thermochemical Tables* , 2nd ed.; National Bureau of Standards: Washington, DC, 1971.

(19) Maintz, S.; Deringer, V. L.; Tchougréeff, A. L.; Dronskowski, R. LOBSTER: A Tool to Extract Chemical Bonding from Plane-Wave Based DFT. *J Comput Chem* **2016**, *37* (11), 1030–1035. https://doi.org/10.1002/jcc.24300.

(20) Nelson, R.; Ertural, C.; George, J.; Deringer, V. L.; Hautier, G.; Dronskowski, R. LOBSTER: Local Orbital Projections, Atomic Charges, and Chemical-Bonding Analysis from Projector-Augmented-Wave-Based Density-Functional Theory. *J Comput Chem* **2020**, *41* (21), 1931–1940. https://doi.org/10.1002/jcc.26353.
